# Identification of forearm skin zones with similar muscle activation patterns during activities of daily living

**DOI:** 10.1186/s12984-018-0437-0

**Published:** 2018-10-29

**Authors:** Néstor J. Jarque-Bou, Margarita Vergara, Joaquín L. Sancho-Bru, Alba Roda-Sales, Verónica Gracia-Ibáñez

**Affiliations:** Department of Mechanical Engineering and Construction, Universitat Jaume I, Avinguda Vicent Sos Baynat, s/n., 12071 Castellón, Spain

**Keywords:** Activities of daily living, Clustering analysis, Electromyography, Electrode placement, Forearm muscles, Functional principal component analysis, Myoelectric prostheses, Rehabilitation, Sollerman hand function test

## Abstract

**Background:**

A deeper knowledge of the activity of the forearm muscles during activities of daily living (ADL) could help to better understand the role of those muscles and allow clinicians to treat motor dysfunctions more effectively and thus improve patients’ ability to perform activities of daily living.

**Methods:**

In this work, we recorded sEMG activity from 30 spots distributed over the skin of the whole forearm of six subjects during the performance of 21 representative simulated ADL from the Sollerman Hand Function Test. Functional principal component analysis and hierarchical cluster analysis (HCA) were used to identify forearm spots with similar muscle activation patterns.

**Results:**

The best classification of spots with similar activity in simulated ADL consisted in seven muscular-anatomically coherent groups: (1) wrist flexion and ulnar deviation; (2) wrist flexion and radial deviation; (3) digit flexion; (4) thumb extension and abduction/adduction; (5) finger extension; (6) wrist extension and ulnar deviation; and (7) wrist extension and radial deviation.

**Conclusion:**

The number of sEMG sensors could be reduced from 30 to 7 without losing any relevant information, using them as representative spots of the muscular activity of the forearm in simulated ADL. This may help to assess muscle function in rehabilitation while also simplifying the complexity of prosthesis control.

## Background

The forearm and the hand are connected by the wrist, together forming a functional unit in which there are about 30 muscles that work in concert in a highly complex way [1], allowing a wide range of activities to be performed, many of them with a high level of precision. When a disease or injury to any part of the hand or forearm take place, our manipulation and grip capacities are reduced and, therefore, carrying out the most common activities in our daily life can become a serious problem.

Surface electromyography (sEMG) is a non-invasive approach that allows both structural and functional characterization of the neuromuscular system, as well as making it possible to quantify variations of this system in different situations. sEMG is applied in many fields such as motor control of human movement, myoelectric control of prosthetic and orthotic devices, and rehabilitation [2–5]. In rehabilitation, sEMG has been used to monitor the effects of rehabilitation techniques [5, 6], rehabilitation devices [7], and therapeutic exercises [8]. In this line, some studies have proposed the addition of sEMG analysis to clinical assessments to provide quantitative measures of therapy outcomes for people with motor impairment and physical disability in the upper limb, such as stroke [9] or hemiplegia [10]. In those studies, the amplitude of the sEMG signal is used to provide quantitative measures, as patients have a reduced neuromuscular amplitude [5, 9, 11]. However, there is no consensus on the muscles or areas of the forearm that must be measured in each case. This fact may lead to the recording of repeated muscle activity information and/or missing information about some muscles.

The central nervous system must control a structure that is in general vastly more complex than necessary to execute any particular task. In this sense, muscular activation patterns or muscular synergies [12, 13] appeared as a possible way to allow the analysis of muscular control during task execution. Some studies have described muscle patterns or synergies during certain postures [14], grasps [15] or hand movements [16, 17], during particular actions [18–22], and for individually tailored rehabilitation protocols [23]. Examining whether muscle synergies change following rehabilitation may provide an assessment of interventions that improve patients’ motor function and therefore their ability to perform activities of daily living (ADL). A patient’s muscle synergy profile may allow clinicians to treat motor dysfunctions more effectively by organizing patients into subclasses and tailoring the treatment to each patient’s specific deficit [24].

Myoelectric prostheses are usually controlled with sEMG [25–27]. However, one quarter to one third of all amputees reject self-powered prostheses [28] because they feel a non-close-to-natural operation [29] that leads to long and discouraging training periods. A deeper knowledge of the activity of the forearm muscles during different functional tasks could help in the development of more intuitively controlled prostheses, capable of replicating the intact hand movements by using sEMG signals from the residual muscles, similarly to when they had their own intact hand. In this sense, identification of repeatable patterns of muscle activity (muscular synergies) across multiple muscle sites rather than relying on independent EMG signals has been shown to enable more natural, reliable control of myoelectric prostheses [30]. However, the electrodes are usually placed on the remaining muscles of the forearm regardless of the muscle’s pre-amputation role [31], favoring a non-close-to-natural operation. To overcome this hindrance, some authors have studied the accuracy of pattern recognition of myoelectric control, by evaluating the number and locations of sEMG channels [30, 32, 33]. Daley et al. [30] distributed the electrodes around the whole forearm in a grid formation during twelve wrist and hand motions and five different grasps in healthy and amputee subjects. Their results suggest that most of the neural information available could be extracted with a greatly reduced number of electrodes. However, the optimal location of electrodes and their consistency across subjects were not reported, and the tasks used for the analysis were not representative of ADL. Upper limb tasks should be goal-oriented and of a standardized nature to obtain consistent performance [34]. Some previous works have evaluated the performance of similar SEMG classification systems by using the NINAPRO database [35–37]. However, the sEMG data contained in this database was recorded while performing non-goal-oriented actions. Clinical tests, such as the Sollerman Hand Function test (SHFT) [38], are often used to evaluate and track functional recovery of the upper extremity, providing insight into functional performance. These tests typically evaluate this functional performance using tasks simulating ADL.

In this work, we recorded sEMG activity from different areas of the whole forearm skin during the performance of the ADL included in the SHFT, a standardized test for hand function evaluation. The aim was to identify skin zones with similar muscle activation patterns in order to determine the minimum number of electrodes required to characterize the muscle activity during simulated ADL without losing any relevant information.

## Methods

### Subjects and tasks

Six able-bodied subjects (3 males and 3 females, with average (SD) age of 34.5 (8.2) years) gave their informed written consent before participating in this study, approved by the ethics committee of our University.

**Fig. 1:**
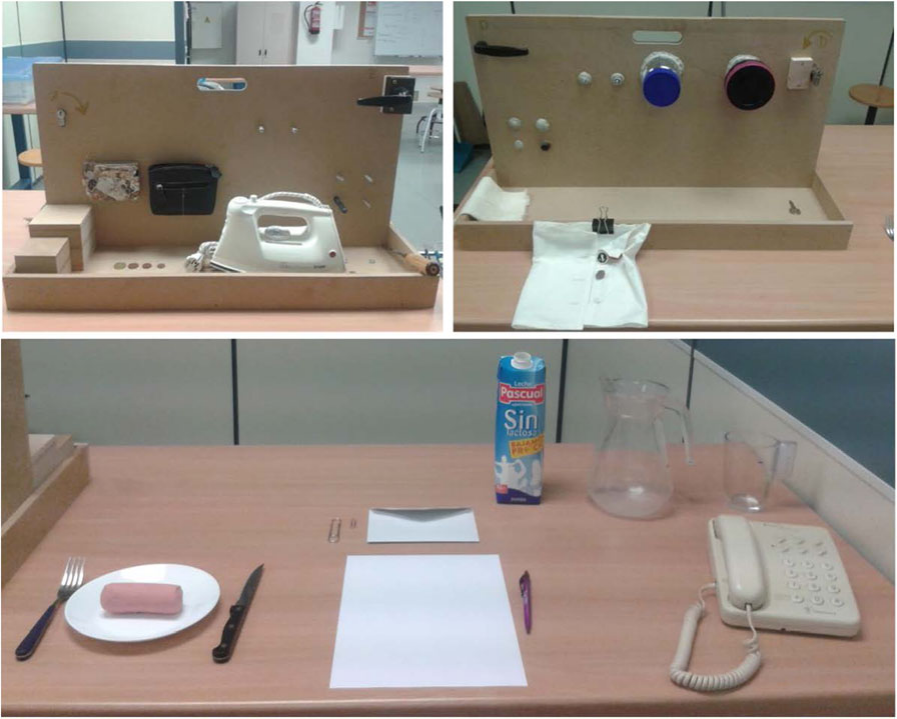
Sollerman Hand Function Test configuration

Twenty-one simulated ADL were selected, all of them tasks included in the SHFT. Some of the simulated ADL were adapted in order to ensure their repeatability. The activity 14 from the original SHFT was separated in two different activities. In the first one, the piece of paper was folded (A14) and in the second one the folded paper was inserted into an envelope (A15). Others were slightly adapted, for example, reducing the number of objects to be manipulated (just one zipper, one coin, one button, etc.) or indicating the direction and the degrees to be turned in each case (Fig. 1, Table 1). Each simulated ADL started and ended with the body and arms in the same posture (arms relaxed at the side of the body, in the case that the person was standing, or arms resting in a relaxed position on the table if they were sitting). Precise instructions were provided for each task by the researcher administering the SHFT, including details such as the angle of rotation of the key (A1), the position of the coin (A2 & A4), the angle of rotation of the door handle (A18) or the amount of water to be poured (A20). The subjects could practice each task as many times as necessary in advance so as to become familiar with its performance before the sEMG recording.

**Table 1:** Description of the standardized ADL

ADLs	DESCRIPTION
A1	Take a key, insert it in a lock, turn it counter-clockwise 180° and leave the key in
A2	Collect a coin and insert it into a change purse
A3	Open and close a zipper
A4	Remove the coin from the change purse and leave it on the table
A5	Catch and move two wooden cubes of different sizes
A6	Lift and move an iron from one marked point to another
A7	Take a screwdriver and turn a screw clockwise 180° with it
A8	Take a nut and bolt and turn the bolt until it is completely inserted inside the nut
A9	Unscrew a lid and leave it on the table
A10	Pass a button through a buttonhole with the help of both hands
A11	Take a knife with the right hand and a fork with the left hand and split a piece of clay (sitting)
A12	Take a bandage and put it on your left arm until the elbow
A13	Pick up a pen from the table, write the Spanish word “SOL” and put the pen back on the table (sitting)
A14	Fold a piece of paper with both hands and insert it into a box
A15	Insert the folded paper (from A14) into an envelope and leave it on the table
A16	Take a clip and put it on the flap of the envelope
A17	Pick up the phone, put it to your ear and hang up the phone
A18	Turn a door handle 30°
A19	Pour 1 L of water from a carton into a jug
A20	Pour water from the jug into the glass up to a marked point
A21	Pour the water from the glass back into the jug

### Electrode placement

**Fig. 2:**
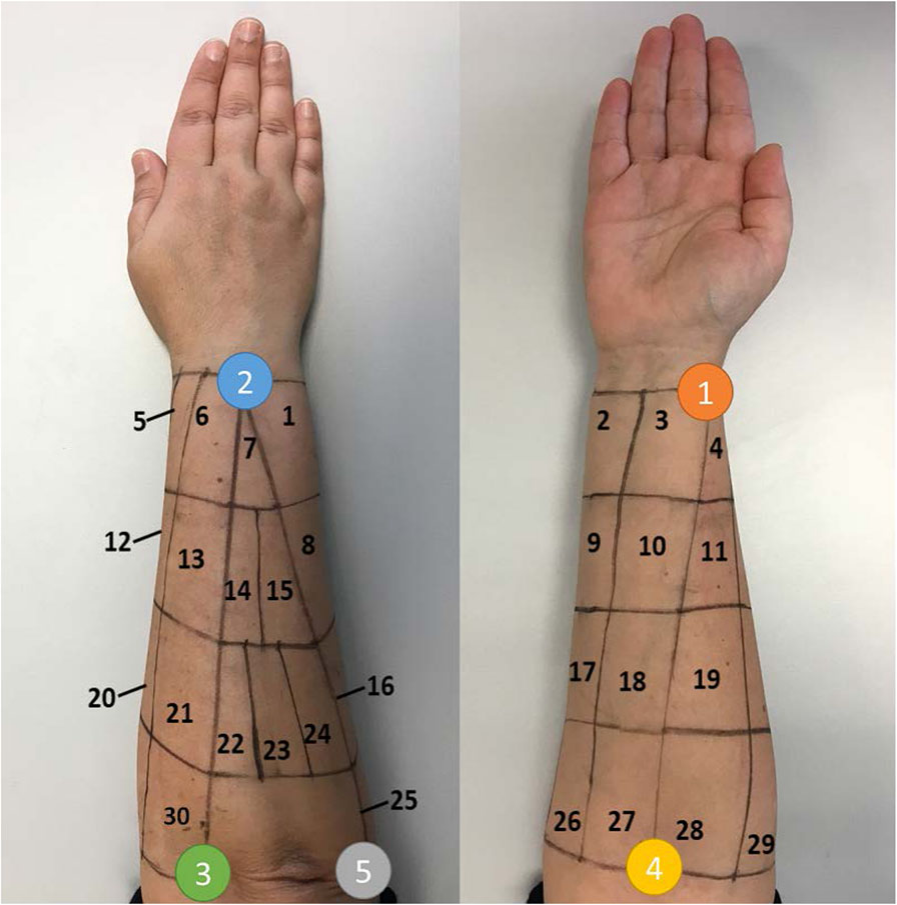
Grid with the 30 spots for sEMG recording, and anatomical landmarks used

A grid was drawn on the forearm, covering its entire surface, by using five easily identifiable and highly reproducible anatomical landmarks (Fig. 2, Table 2). The grid defined 30 different spots (Fig. 2) and was drawn with the subject sitting comfortably, the elbow resting on the table, the arm flexed 90° with respect to the forearm, and the palm of the hand facing the subject. First, three longitudinal lines were drawn joining the anatomical landmarks 1–4, 2–3 and 2–5, and two transverse lines joining landmarks 1–2 and 3–4-5. Each of the longitudinal lines was divided into four equal parts that were used to draw the internal transverse lines of the grid. Transverse lines 1–2 and 4–5 in the cubital region were divided into three equidistant parts, and the same was applied to lines 1–2 and 4–3 in the radial region. These subdivisions were used to draw the corresponding longitudinal lines. Finally, the area formed by landmarks 2–3-5 was divided so that there were three equal spots in the most proximal region (spots 22, 23 and 24), a single spot in the most distal region (spot 7), and two spots in the intermediate region (spots 14 and 15), as shown in Fig. 2. The sensors were placed in the centre of each area and were set out in a longitudinal direction, since most of the muscles of the forearm are quite aligned to it [1]. Before placing the electrodes, the hair was removed by shaving and the skin was cleaning by using alcohol.

**Table 2:** Anatomical landmarks

Number	Landmark
1	Styloid processes of the radius
2	Ulna head
3	Medial epicondyle of the humerus
4	Center point of the elbow
5	Humeral lateral epicondyle

## Data acquisition

**Fig. 3:**
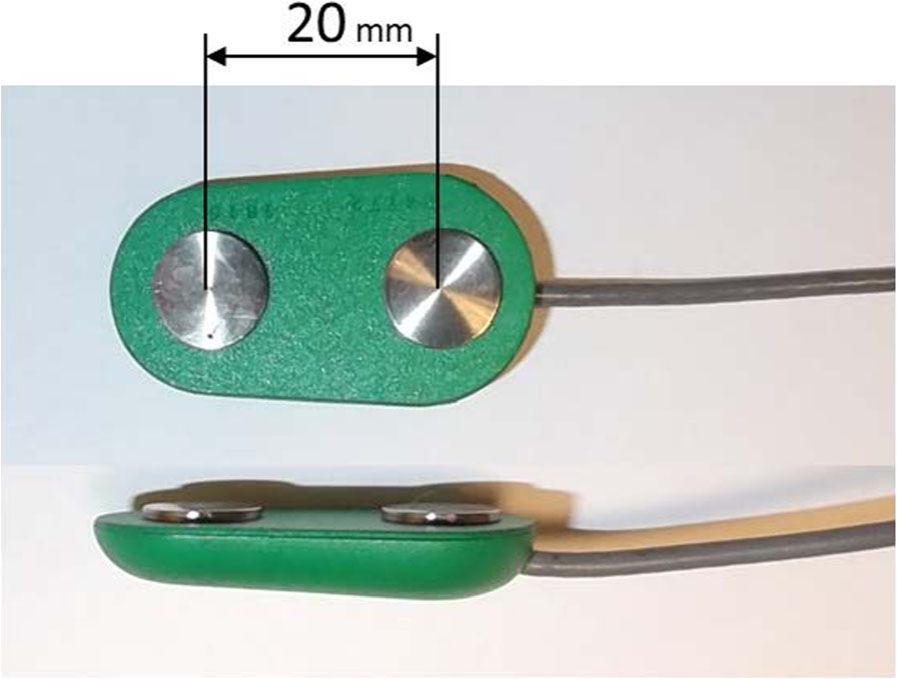
sEMG Electrodes (SX230) used: integral dry reusable electrodes with a gain of 1000, bandwidth between 20 Hz – 460 Hz and noise less than 5 μV

Muscle activity was recorded with an 8-channel sEMG Biometrics Ltd. device, with a sampling frequency of 1000 Hz. Technical information of the electrodes is shown in Fig. 3. Recording sEMG from the 30 spots of the grid required distributing the measurements over five different sessions. The spots chosen for each session (the same for each subject) were selected to be as widely distributed as possible, so that electrodes were placed in spots in not close to each other. Each simulated ADL was recorded twice within each session. It was also checked that the duration of the same simulated ADL was similar in each session. In order to check the repeatability of the simulated ADLs among sessions, all sessions recorded sEMG from spot 30.

The sEMG records were filtered with a 4th-order bandpass filter between 25 and 500 Hz, rectified, filtered by a 4th-order low pass filter at 8 Hz, and smoothed by Gaussian smoothing. To determine muscle activity, sEMG records were normalized with the maximal values from seven records of maximum voluntary contraction (MVC), measured in each session: flexion and extension of the wrist, flexion and extension of the fingers, pronation of the forearm, ulnar deviation of the wrist, and elbow flexion. MVCs for the different movements were recorded without the use of any external device. In a comfortable posture, the subjects were asked to exert maximum effort (recorded with a dynamometer) without the help of other muscles than those of the forearm and hand. In addition, the beginning and end of the sEMG records were cut to eliminate the first and last moments in which muscle activity did not exceed 5% in any of the spots. Finally, to make records comparable in duration, all the records were interpolated to 1000 data.

## Data analysis

i) ADL repeatability: The confidence intervals (CI) of the 10 muscular activity records (2 repetitions × 5 sessions) of spot 30 were computed for each simulated ADL (1000 values of CI per ADL × 6 subjects). Statistics per ADL were considered for the analysis of repeatability.

ii) Reduction of temporary muscle activity data: The data from all repetitions at each spot for each simulated ADL were averaged, and used in the subsequent analysis, so that a total of 3780 signals (21 ADL × 6 subjects × 30 spots) of 1000 temporary data were considered. The Principal Component Analysis (PCA) was one of the first methods adapted to functional data. The fundamental idea of this extension is to conserve all the benefits of the PCA as a tool for the reduction of the dimension, conditioning them to functional data. Thus, the Functional Principal Component Analysis (FPCA) [39] could provide a basis for creating a new set of variables that incorporate the character and nature of the original functions into a smaller number of new variables. In this case, FPCA was applied to condense the temporary muscle activation data into a smaller number of parameters, in order to make the comparison of temporal patterns feasible. First, for each subject, the signals for the 21 simulated ADL from each spot were concatenated. Subsequently, each of these signals was normalized to 1 using their maximum value, in order to keep only the temporal patterns to be analyzed. Then, the data of all the subjects were concatenated. In this way, the 3780 signals were transformed into 30 functions of 126,000 data, one function per spot. FPCA was applied then to these 30 functions, extracting the functional principal components (FPCs) that explained at least 90% of the cumulative variance. Each of the 30 original functions can be expressed therefore as a common mean function plus a linear combination of the FPCs, with specific coefficients for each original function, hereinafter called reduced variables (RVs), as they condense the 126,000 data into a small number of coefficients.

iii) Similarity of patterns of muscle activation: Conglomerate or Hierarchical clustering analysis [40] is a multivariate technique that allows the classification of elements in groups or clusters, so that each element is very similar to those in its own conglomerate according to some specific selection criteria. In this case, Hierarchical clustering analysis, with the Euclidean distance as the distance criterion and Ward’s method as the linkage criterion, was applied to the RVs from the 30 spots to group spots with similar muscle activation patterns. The resulting dendrogram with the spots organized in branches was displayed, and the desired number of clusters was identified by observing the distances in each step. When the distance between the clustered groups in a step becomes high in comparison to the previous steps, the elements or clusters grouped are not so close and so the grouping of the previous step may be more appropriate. The resulting groups of spots with a similar activation pattern were described. Additionally, root mean square (RMS) values of all signals measured at each spot were computed for analysis of representativeness, in order to choose a specific spot as being representative of each group.

## Results

**Fig. 4:**
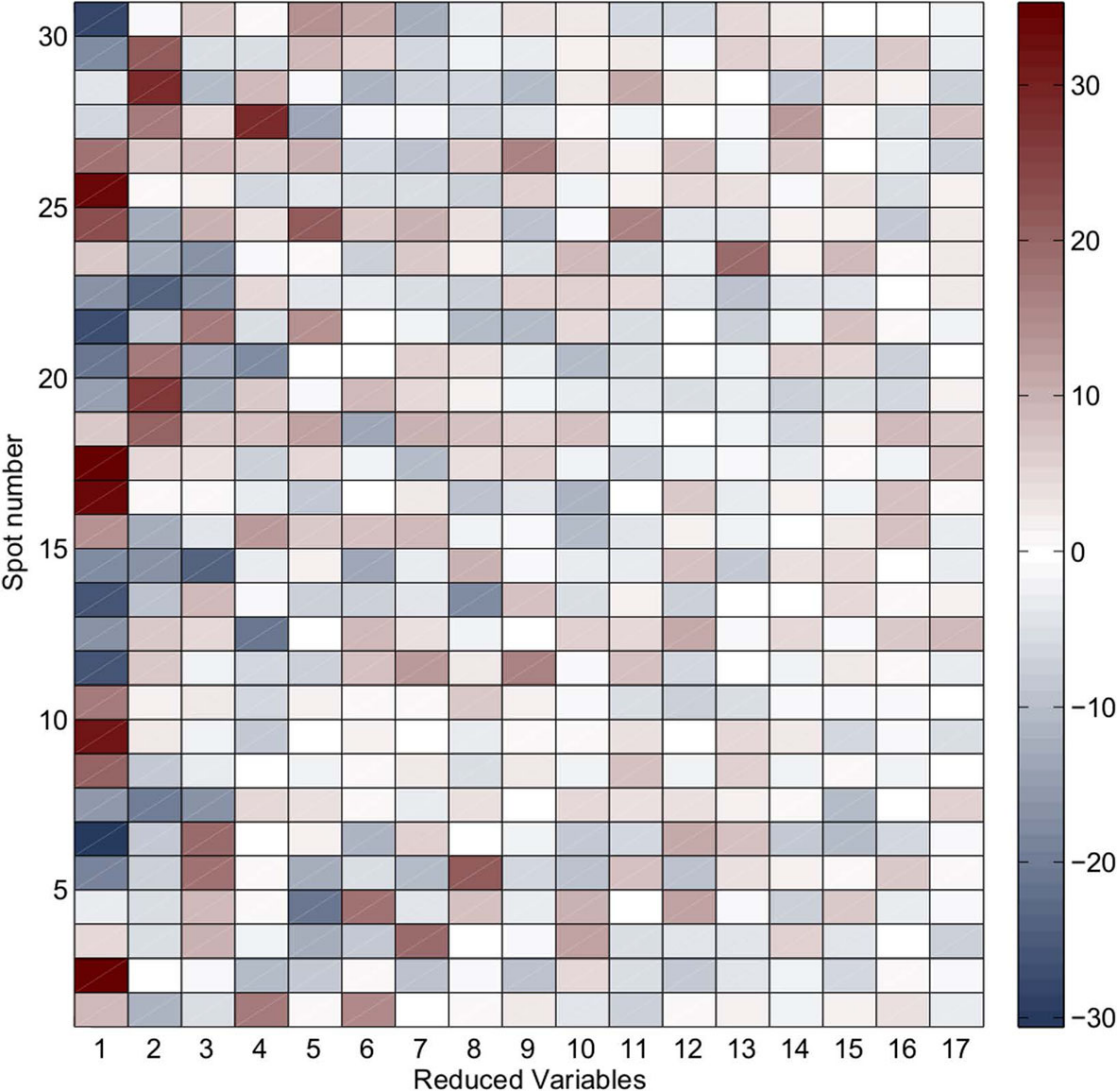
Values of the 17 RVs for each of the 30 spots. Positives values are displayed in red and negative values in blue

**Fig. 5:**
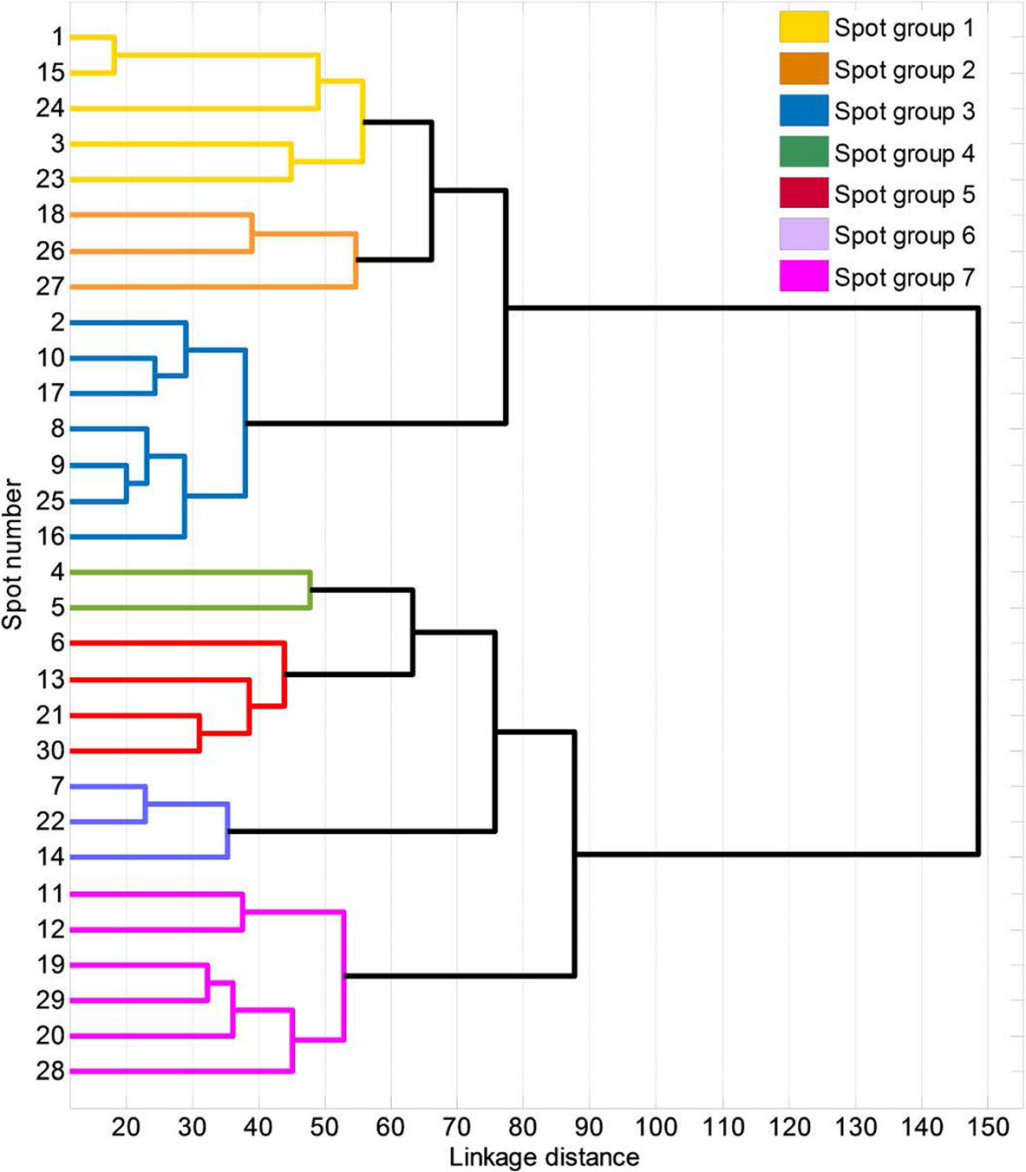
Dendrogram obtained from the hierarchical clustering. The seven groups chosen are displayed in different colours

**Fig. 6:**
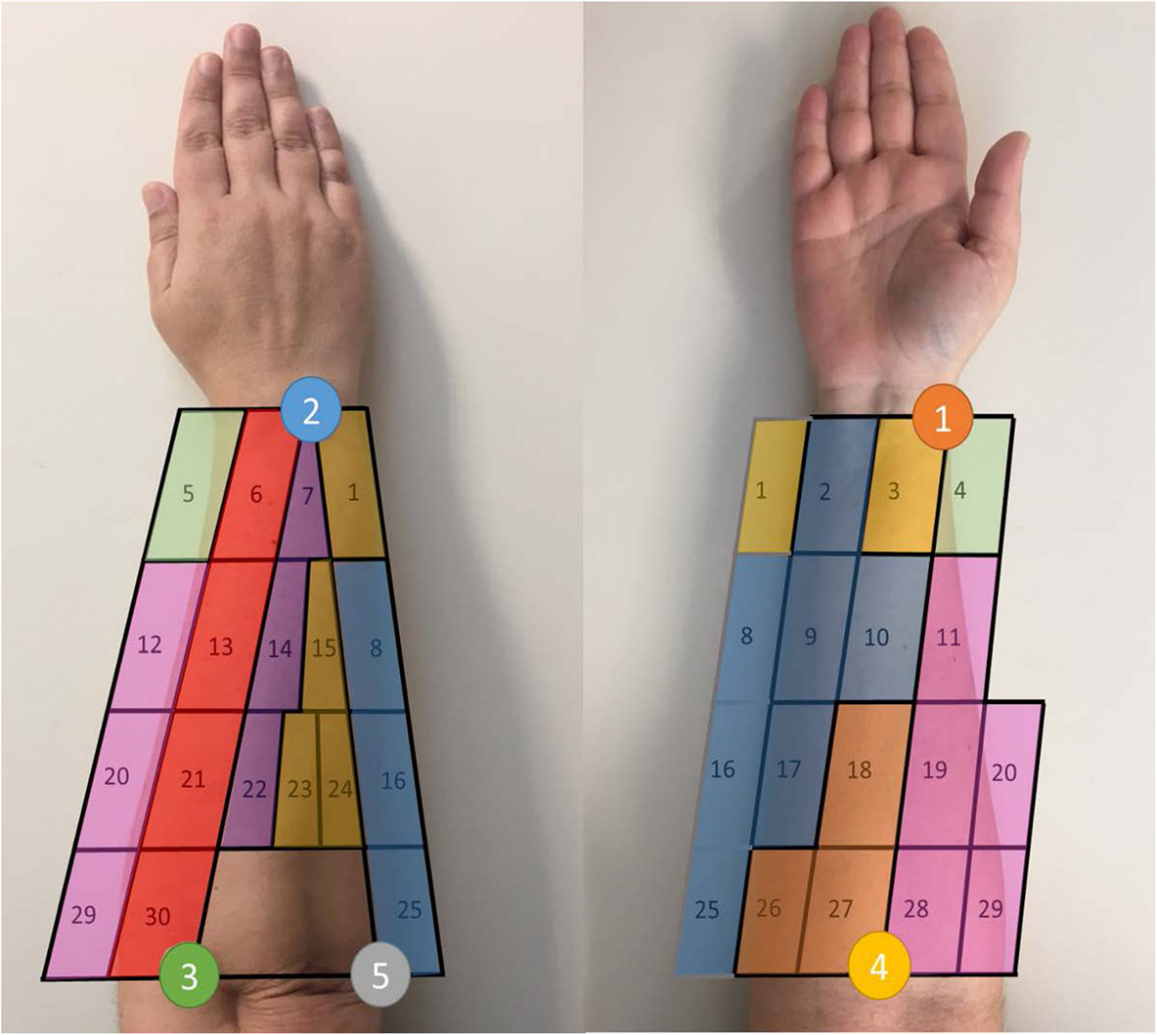
Resulting groups of spots with similar activation patterns

i) ADL repeatability: Statistics of the CI of the muscular activity records at spot 30 for each simulated ADL are shown in Table 3 as a percentage of MVC. In general, the results showed a good level of repeatability of the different simulated ADL, with mean CI values of 3.33% of muscular activity and SD values of 2.22%. The most unfavorable results were for ADL 9, with mean CI of 6% (SD 4.2%) of MVC.
ii) Reduction of temporary muscle activity data: 17 FPCs explained 91% of the total variance. RVs are displayed in Fig. 4 as a rectangular array of colored cells defined by the values of the RVs.
iii) Similarity of patterns of muscle activation: Fig. 5 shows the dendrogram obtained from the hierarchical clustering with the distance between grouped elements. The dendrogram shows different possible groupings, depending on the linkage distances. The resulting groups for the case of seven clusters are displayed with different colors in Fig. 6.

**Table 3:** CI Statistics: mean and standard deviation (SD)

ADL	mean	SD
1	2.6%	2.3%
2	1.9%	1.4%
3	1.7%	1.4%
4	3.6%	3.2%
5	4.3%	2.5%
6	5.0%	3.1%
7	3.9%	2.6%
8	2.9%	2.8%
9	6.0%	4.2%
10	2.7%	1.6%
11	4.1%	2.2%
12	3.9%	2.7%
13	2.9%	1.1%
14	3.1%	1.6%
15	3.7%	2.5%
16	2.1%	1.3%
17	3.8%	2.3%
18	2.7%	1.5%
19	2.2%	2.1%
20	4.0%	2.6%
21	2.9%	1.6%

**Fig. 7:**
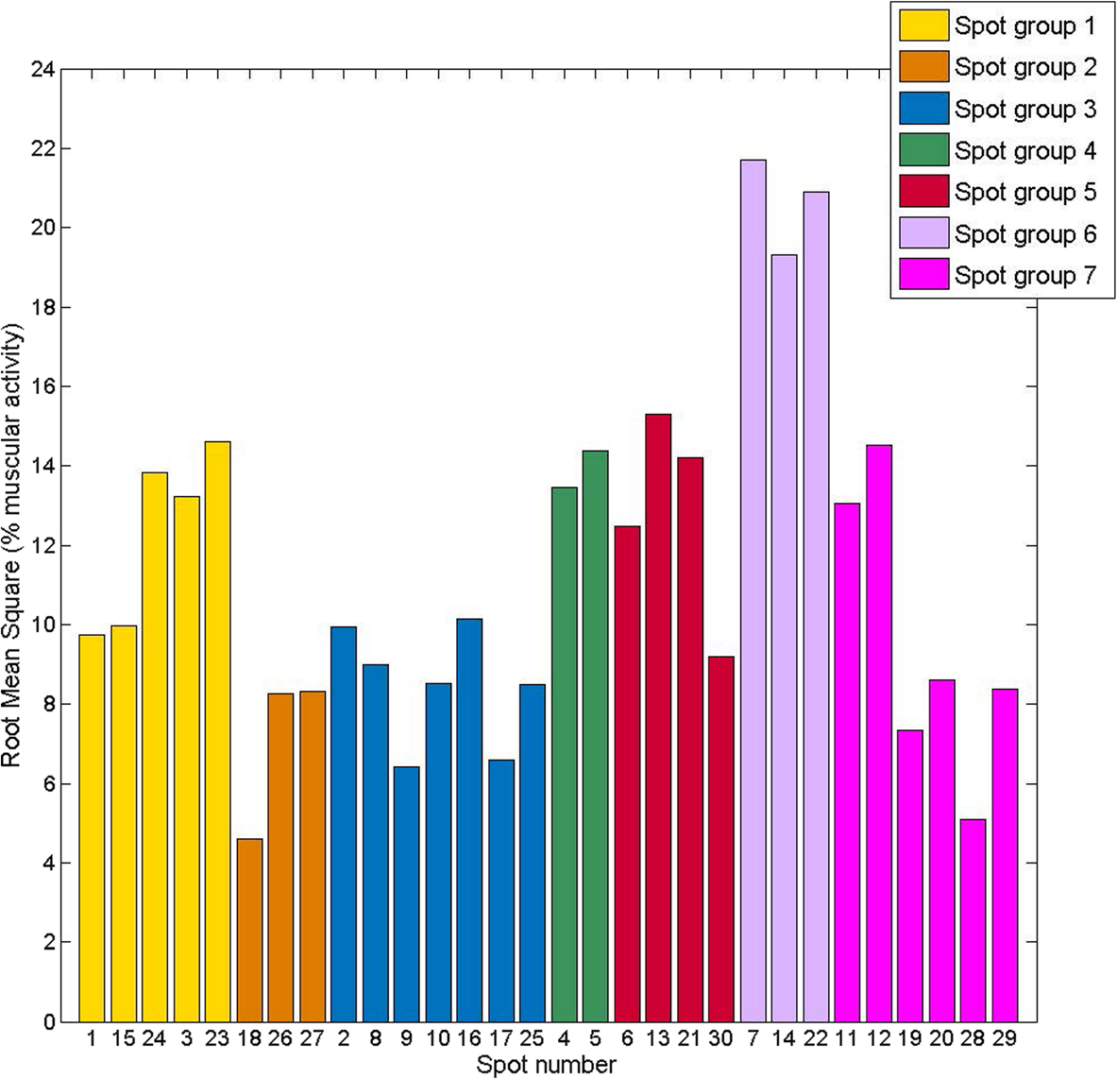
RMS of the muscular activity values of each spot. The bars are colored according to the resulting groups

Figure 7 shows the RMS values of the muscular activity measured at each spot, to be considered when choosing a specific spot as representative of a group. Spots 18 and 28 show a low level of RMS values compared to the other spots, reaching less than 5% of RMS of the muscular activity.

## Discussion

In this work we identified forearm areas with similar muscle activation patterns by means of FPCA and cluster analysis, which could be used to characterize the muscle activity during relevant simulated ADLs. Consequently, one of the main contribution of the proposed approach is the focus on goal-directed actions.

The tasks used in this study were taken from the SHFT. The selection of the tasks was originally based on the percentage use of the most common handgrips during ADL and hence reflects an accurate representativeness of hand function in day-to-day life. Muscle activity on 30 different forearm spots was obtained for each of the 21 representative simulated ADLs by merging sEMG signals recorded in five different sessions This was possible because of the high repeatability observed for the simulated ADLs considered (mean CI values at spot 30 of 3.33% of MVC, SD 2.22%), thanks to the proposed standardization. Unscrewing a lid and leaving it on the Table (ADL 9) was the least repeatable activity, with a mean CI value of 6% of MVC.

Seventeen RVs explained 91% of the total variance. This new set of variables provided the same information but in a more easily interpretable way: each of the 30 original functions of 126,000 temporary data was expressed as a common mean function plus a linear combination of 17 FPCs, with specific coefficients (RVs) for each original function. These 17 RVs obtained from the FPCA have been used in a hierarchical cluster analysis to group spots with similar activation patterns.

From the observation of the distances in each step in the hierarchical cluster analysis, seven groups may be established based on the moment in which the distance between the clustered groups in a step becomes high in comparison to the previous steps. Therefore, we could reduce the number of EMG sensors from 30 to 7 without losing any relevant muscle activity information during the performance of simulated ADL. This is very interesting, as there are 20 muscles superimposed on each other in the forearm, thus making it practically impossible to isolate the sEMG signal of each muscle. Protocols of muscular function assessment of the forearm in rehabilitation can benefit from this. The measurement of the amplitude of the sEMG signals from the seven resulting groups may provide a reasonably complete quantitative picture of the patient’s rehabilitation outcome. Furthermore, given that the number of available electrodes could be limited and that a smaller number of EMG signals may simplify the assessment, knowing the similarity of the sEMG signals with different levels of hierarchy, as provided with the method used, is also quite interesting. This also applies for prosthesis control, where the number of EMG signals to be used is limited by the complexity of the controller.

When observing the dendrogram obtained from the cluster analysis, different numbers of clusters could be chosen. Two main hierarchy levels can be observed: one involves the flexor muscles of the wrist and digits (spot groups 1 to 3), and the other includes their extensor muscles (spot groups 4 to 7). In the next hierarchy level, flexors bifurcate into wrist flexors (groups 1 and 2) and digit flexors (group 3); it is known that digit flexors also contribute to wrist flexion [41]. And something similar occurs for extensors, which bifurcate into digit extensors (groups 4 and 5) and wrist extensors (groups 6 and 7). Therefore, in the case of using two signals for prosthesis control, it would be logical to consider the two main hierarchy levels for an intuitive control in carrying out ADL, associating the sEMG signals to the performance of flexion and extension movements of the prosthesis. In the case of using four control signals, we could use the next hierarchy level described, differentiating between hand and wrist flexors and extensors. However, the higher the number of signals is, the more difficult it will become to separate them, and using groupings that are different from those resulting from the dendrogram will not be so intuitive.

If we were to divide the classification a step further we would get the seven groups described in the results section: Group 1 is defined by spots 1, 3, 15, 23 and 24. Anatomically, it could be recording mainly the muscle activity of the flexor carpi ulnaris (FCU). The FCU is a very powerful muscle that acts as a flexor and ulnar deviator of the wrist [41], being responsible for stabilizing it during activities such as slicing meat and using a hammer. Group 2 is defined by spots 18, 26, and 27, and could be recording the muscle activity of the flexor carpi radialis (FCR) and palmaris longus (PL). Both FCR and PL are reported as wrist flexors, and FCR also as a radial deviator [41]. Group 3 is defined by spots 2, 8, 9, 10, 16, 17 and 25, and could be recording the muscle activity of the digit flexor muscles: flexor digitorum superficialis (FDS) and profundus (FDP), and flexor pollicis longus (FPL). The FDS and FDP muscles are finger flexors [42], and are located on the same forearm area, but at different depths. FPL is a flexor of all three joints of the thumb, and is the only thumb interphalangeal (IP) joint flexor [43]. Group 4 is defined by spots 3 and 4, placed next to the thumb. This group could be recording the muscle activity of the abductor pollicis longus (APL), and extensor pollicis longus (EPL) and brevis (EPB). EPB supports the extension of the metacarpophalangeal (MCP) joint of the thumb, APL participates in the abduction and extension of the thumb carpometacarpal (CMC) joint, and EPL extends the three thumb joints, and adducts the thumb CMC joint [41]. Moreover, all these muscles contribute to wrist radial deviation [41]. Group 5 is defined by spots 6, 13, 21 and 30, and could be recording the muscle activity of the extensor digitorum communis (EDC). The EDC is the primary extensor of the MCP joints of the fingers, although it also contributes to the extension of the proximal (PIP) and distal (DIP) interphalangeal joints of the fingers [41]. Group 6 is defined by spots 7, 14 and 22, and could be recording the muscle activity of the extensor carpi ulnaris (ECU). ECU has the largest moment arm for ulnar deviation and plays an important role in supporting the distal radioulnar joint [43], the joint that enhances the manipulating skills of the hand [44]. Finally, Group 7 is defined by spots 11, 12, 19, 20, 28 and 29, and could be recording the muscle activity of the brachioradialis (BR), pronator teres (PT), extensor carpi radialis brevis (ECRB) and longus (ECRL). The main role of the ECRB and ECRL is wrist extension and radial deviation [43]. However, some studies suggested that ECRL and ECRB could have pronation and supination elbow moments [45]. BR and PT contribute to elbow flexion as well as forearm pronation and supination. Moving forward at the hierarchical level would not make sense, since we would be selecting groups of points where the distance between the spots grouped in the next step is low compared to the previous steps, and the number of sEMG signals to be used is limited.

Therefore, we propose using seven groups of spots for characterizing the muscular activity of the forearm during simulated ADL, in order to substantially reduce the number of spots to be registered, and to maintain muscular-anatomical coherence. The signals from these seven spots would be related to seven different movements: (group 1) wrist flexion and ulnar deviation; (group 2) wrist flexion and radial deviation; (group 3) digit flexion; (group 4) thumb extension and abduction/adduction; (group 5) finger extension; (group 6) wrist extension and ulnar deviation; and (group 7) wrist extension and radial deviation.

When assessing muscle function in rehabilitation, some spots may be unavailable because of the simultaneous usage of other equipment, such as sensors for kinematics measurement. When choosing the representative spots of each group to be recorded from among those available, the percentage of muscle activity measured (Fig. 7) should be taken into account. Selecting the spots with the highest percentage of muscle activity may be more reliable, since, at these spots, the muscles recorded could be more superficial or the muscle area recorded could be more centered. In particular, spots with a very low level of muscle activity should be avoided, such as spots 18 and 28. The greatest muscle activity was observed in the most distal part of the forearm (Fig. 7). However, more proximal spots could be chosen, depending on the availability, except for group 4, which is composed of two spots placed in the most distal part of the forearm.

FPCA is one of the most popular multivariate analysis techniques for extracting information from functional data, reducing the dimensions of a data set in which there are a large number of interrelated variables, while still holding as much of the total variation as possible [39]. While FPCA results in dimension reduction, FPCA vector scores can be used for clustering different functions/components using standard clustering methods. Clustering is one of the most frequently used techniques for partitioning a dataset into subgroups that contain instances that are similar to each other while being clearly dissimilar to those of the other groups. In a functional context, clustering helps to identify representative curve patterns and individuals who are very likely to be involved in the same or similar processes. Other methods reported in the literature [46] used for task identification in prosthesis control are based on the segmentation of the EMG signals into a series of windows, in which some commonly used time-domain feature sets (such as Mean Absolute Value or Zero Crossings) are extracted and used for motion classification. Our method is similar, but applied to the entire signal for all tasks and subjects, and extracting from the FPCA the features of the signal that holds as much of the total possible variation and using them for muscular classification.

The EMG recordings have been carried out with an 8-channel sEMG device, which required the repetition of the same activities for each subject in five different sessions to allow the measurement of the high number of spots chosen. Although reproducibility error has been checked to be small for the spot 30 between sessions, the use of high-density surface EMG might be considered in upcoming works.

The current study has been limited to six healthy, able-bodied subjects, and the results could be verified in further studies with a higher number of subjects and including impaired subjects. However, muscular groupings obtained in this study may be used as a first approximation, and may be used as guide for future validation for subjects with hand impairments or amputees.

As future work, studies could be conducted to relate EMG to kinematics during the performance of ADL, by using these seven spots as representative of the muscular activity of the forearm in ADL. A further step would be to evaluate kinematics and muscular synergies during specific functional tasks.

## Conclusions

This study aimed to identify skin zones with similar muscle activation patterns in order to determine the minimum number of electrodes required to characterize the muscle activity during simulated ADL without losing any relevant information. The results indicate that we could reduce the number of sEMG sensors from 30 to 7 and use them as representative spots of the muscular activity of the forearm in simulated ADL. The simulated ADL performed are included in the SHFT, which uses the most common handgrips in day-to-day life. Hence, the simulated ADL chosen in this study may reflect an accurate representativeness of the hand function. This result may help to assess muscle function in rehabilitation as well as simplify the complexity of prosthesis control.

## Abbreviations

ADL: Activities of daily living
APL: Abductor policis longus
BR: Brachioradialis
CI: Confidence intervals
CMC: Carpometacarpal
DIP: Distal interphalangeal
ECRB: Extensor carpi radialis brevis
ECRL: Extensor carpi radialis longus
ECU: Extensor carpi ulnaris
EDC: Extensor digitorum communis
EPB: Extensor policis brevis
EPL: Extensor policis longus
FCR: Flexor carpi radialis
FCU: Flexor carpi ulnaris
FDP: Flexor digitorum profundus
FDS: Flexor digitorum superficialis
FPCA: Functional principal component analysis
FPCs: Functional principal components
HCA: Hierarchical clustering analysis
IP: Interphalangeal
MCP: Metacarpophalangeal
MVC: Maximum voluntary contractions
PIP: Proximal interphalangeal
PL: Palmaris longus
PT: Pronator teres
RMS: Root mean square
RVs: Reduced variables
SD: Standard deviation
sEMG: Surface electromyography
SENIAM: Surface electromyography for the non-invasive assessment of muscles
SHFT: Sollerman hand function test
